# *In vitro* anti-mycobacterial activity of nine medicinal plants used by ethnic groups in Sonora, Mexico

**DOI:** 10.1186/1472-6882-13-329

**Published:** 2013-11-25

**Authors:** Ramón Enrique Robles-Zepeda, Enrique Wenceslao Coronado-Aceves, Carlos Arturo Velázquez-Contreras, Eduardo Ruiz-Bustos, Moisés Navarro-Navarro, Adriana Garibay-Escobar

**Affiliations:** 1 Chemical Biological Science Department, Biological and Health Sciences Division, University of Sonora, Hermosillo, Sonora, Mexico; 2 Chemical Biological Sciences Department, University of Sonora, Rosales and Luis Encinas, CP 83000 Hermosillo, Sonora, Mexico

**Keywords:** Medicinal plants, Tuberculosis, Alamar blue, Mycobacterium tuberculosis, Ambrosia

## Abstract

**Background:**

Sonoran ethnic groups (Yaquis, Mayos, Seris, Guarijíos, Pimas, Kikapúes and Pápagos) use mainly herbal based preparations as their first line of medicinal treatment. Among the plants used are those with anti-tuberculosis properties; however, no formal research is available.

**Methods:**

Organic extracts were obtained from nine medicinal plants traditionally used by Sonoran ethnic groups to treat different kinds of diseases; three of them are mainly used to treat tuberculosis. All of the extracts were tested against *Mycobacterium tuberculosis* H37Rv using the Alamar Blue redox bioassay.

**Results:**

Methanolic extracts from *Ambrosia confertiflora*, *Ambrosia ambrosioides* and *Guaiacum coulteri* showed minimal inhibitory concentration (MIC) values of 200, 790 and 1000 μg/mL, respectively, whereas no effect was observed with the rest of the methanolic extracts at the concentrations tested. Chloroform, dichloromethane, and ethyl acetate extracts from *Ambrosia confertiflora* showed a MIC of 90, 120 and 160 μg/mL, respectively.

**Conclusions:**

*A. confertiflora* and *A. ambrosioides* showed the best anti-mycobacterial activity *in vitro*. The activity of *Guaiacum coulteri* is consistent with the traditional use by Sonoran ethnic groups as anti-tuberculosis agent.

For these reasons, it is important to investigate a broader spectrum of medicinal plants in order to find compounds active against *Mycobacterium tuberculosis*.

## Background

Tuberculosis (TB) is a chronic infectious disease caused mainly by *Mycobacterium tuberculosis*[1,2]. The World Health Organization (WHO) estimated that almost 9 million new cases and 1.4 million TB deaths (990 000 among HIV-negative people and 430 000 HIV-associated TB deaths), occurred in 2011 [3]. Ninety-five percent of TB cases are produced in underdeveloped countries, 80% of them corresponding to the 15 to 29-year-old group, generating strong socioeconomic problems [4]. Furthermore, the lack of treatment adherence has given rise to antibiotic-resistant *M. tuberculosis* strains. The resistance is classified into two groups: the multidrug-resistant TB (MDR-TB), which does not respond to the first-line standard treatment, and the extensively drug-resistant TB (XDR-TB), which occurs when resistance to second-line drugs develops [5]. According to the new 2012 WHO report on surveillance and response to MDR-TB and XDR-TB, an estimate of 310 000 (range, 232 000–400 000) MDR-TB cases occurred among recorded pulmonary TB patients in 2011, with 84 countries reporting at least one case of XDR-TB [3]. This emphasizes the need to search for new drugs against tuberculosis [6,7].

According to the National Epidemiological Surveillance (SINAVE), Mexico has shown a reduction in TB incidence and has been considered as a medium security risk region by WHO. Nevertheless, 17 000 new cases and approximately 2000 deaths each year are reported in Mexico [8,9].

Mexico possesses a great geographic diversity, and also has one of the richest flora on the planet, compared with that in Malaysia and some regions in central and South America [10]. Sonora is located on the northwestern region of Mexico and is the second largest state of the country, characterized by more than 3000 plant species widely known and used by the local ethnic groups: Yaquis, Mayos, Seris, Guarijíos, Pimas, Kikapúes, and Pápagos [11]. These groups use herbal remedies in their traditional medicine for cultural reasons, as well as due to the inaccessibility to medical services and/or their expectative of resolving pathologies considered incurable or inoperable by modern medicine [10,11]. Since no report exists yet on studies showing the anti-tuberculosis activity of Sonoran plants, the objective of the present research was to evaluate the potential anti-mycobacterial activity of medicinal plants used by the Sonoran ethnic groups for the treatment of tuberculosis and other diseases.

## Methods

### Plants and extracts preparations

#### Plants collection

Nine plants were selected based on their traditional use by Sonoran ethnic groups for tuberculosis or symptom-related diseases, such as cough, fever, lack of appetite, and weakness or caquexia (Table 1). Plants were collected from wild environments located in the surrounding area of Hermosillo, Sonora, and *Phoradendrom californicum* was collected in a zone located 80 km South of Hermosillo; both collecting areas belong to the Sonoran desert ecosystem [12]. The aerial parts, fruits, and/or flowers were obtained and handled separately. The plants were authenticated at the Herbarium of the University of Sonora by Professor Jesús Sánchez-Escalante, where voucher specimens were deposited.

**Table 1:** Plants used against tuberculosis and symptom-like diseases by Sonoran ethnic groups

**Scientific name (Family)**	**Common name**	**Traditional use**	**Part of plant used**	**Ethnic group usage**	**Voucher**
*Ambrosia confertiflora* DC. (Asteraceae)	Estafiate, istafiate, chi’ichibo, chibchibo	Intestinal parasites, stomach ache, fever, lack of appetite, menstrual symptoms	Leaf and branches	Yaquis, Mayos	17401
*Ambrosia ambrosioides* (Cav.) W.W. Payne (Asteraceae)	Chicura, nagua, toiwe, jupu, joroguo	Wounds, sores, placental expulsion, menstrual symptoms, hair diseases	Leaf and root	Mayo, Ópata, Seri, Yaqui	17404
*Guaiacum coulteri* A. Gray (Zygophyllaceae)	Guayacán, mocni	Dysentery, syphilis, rheumatism, tuberculosis, fever	Fruit, flowers	Seris	17400
*Acalypha californica* Benth. (Euphorbiaceae)	Cupper leaf, cancer weed, bajer cupaj buy	Mouth, stomach, intestine and skin cancer	Branches	Pimas	17402
*Schinus molle* L. (Anacardiaceae)	Pirul	Tuberculosis	Branches	Mayo	17399
*Vallesia glabra* (Cav.) Link (Apocynaceae)	Citabaro, huevito citabaro, otatabe, tonóopa, timóopa, palo verde	Eye inflammation, measles, rheumatism, muscular pain	Fruit and Leaves	Mayo, Yaqui, Seri	18150
*Baccharis salicifolia* (Ruíz & Pavon) Pers. (Asteraceae)	Batamote, jarilla, pasmo weed, bachomo, guachomo, uubachomo	Obesity, anti-contraceptive, hemorrhoids, hair disease, rabies, wounds, digestive disorders	Branches	Mayo, Opata, Seri	17398
*Phoradendrom californicum* Nutt. (Loranthaceae)	Toji, chipchia, aaxt	Diarrhea, stomach polyps, venereal disease, “in-body” diseases	Branches, leaves and cortex	Mayo-Guarijíos, Seri	17405
*Acacia farnesiana* (L.) Wild (Fabaceae)	Huizache, vinorama, Kuká	Throat and digestive tract inflammation, cold, diarrhea, typhoid, wounds, swollen spleen, weak heart, tuberculosis, and headache	Gum, flower, seed, leaves, cortex and root	Mayo-Guarijíos	17406

### Preparation of methanolic extracts of collected plants

One-hundred grams of dry samples were macerated and kept in 1 L methanol at room temperature for one week, with occasional stirring. Solids were filtered out and the extracts concentrated by evaporation under reduced pressure at 40°C in a Yamato RE300 rotator evaporator [13]. For their use in the susceptibility testing, working solutions of the methanolic extracts were prepared in Middlebrook 7H9 broth containing 20% DMSO, at four times the maximum desired testing concentration. All solutions were sterilized by filtration through a 0.22-μm pore size nitrocellulose membrane (Millipore). Final concentration of DMSO in the assay was ≤5%, which does not produce mycobacterial toxicity (internal control).

### Preparation of organic extracts of plants with anti-mycobacterial activity

The plant whose methanolic extract exhibited anti-mycobacterial activity at ≤ 200 μg/mL was further extracted with dichloromethane, chloroform, and ethyl acetate, with a similar protocol to that used for methanolic extract preparation. This extraction was done non-sequentially.

### Mycobacterium tuberculosis

*Mycobacterium tuberculosis* strain H37Rv (sensitive to streptomycin, isoniazide, rifampicin, ethambutol, and pyrazinamide) was provided by the Institute of Epidemiological Diagnosis and Reference of the Mexican Ministry of Health, and was stored and handled at the State of Sonora’s Public Health Laboratory.

### Preparation of *Mycobacterium tuberculosis* inoculum

An initial inoculum was prepared from a solid *M. tuberculosis* culture, until it reached the log growth phase (approximately 12 days). The bacterium was then transferred to a sterile vial containing five glass pearls (3 mm) and 8 mL of sterile 0.85% saline solution. The bacterial suspension was disaggregated by agitation using a Genie II vortex, and left to stand for 15 min at room temperature. The supernatant was then adjusted, using the 1 McFarland standard, to obtain a bacterial concentration of 3.0 × 10^8^ CFU/mL. The working solution was a 1:25 dilution of this suspension, in Middlebrook 7H9 broth supplemented with 0.2% (v/v) glycerol and 10% (v/v) OADC (oleic acid, albumin, dextrose, and catalase enrichment; Becton Dickinson) for the *in vitro* anti-mycobacterial activity assay.

### *In vitro* anti-mycobacterial assay

To evaluate the activity of organic extracts, the redox Alamar Blue® (AbD Serotec) microplate assay (MABA) was carried out as described by Franzblau and collaborators [14]. The assay was carried out on 96-well polystyrene flat bottom plates with low evaporation cover lids, in which the *M. tuberculosis* inoculum was added to supplemented Middlebrook 7H9 broth medium, and mixed with the extracts at different concentrations. In order to prevent excessive evaporation, sterile distilled water was added to the perimetral wells. Rifampicin (Sigma Aldrich) was used as control. Next, 50 μL of fresh Alamar Blue-10% Tween 80 (Sigma Aldrich) mixture (1:1) were added to each well. The microplate was sealed with parafilm and incubated for 48 hours at 37°C. Finally, MIC was calculated and defined as the lowest extract or antibiotic concentration at which no color change of the indicator was evident. Each extract was tested in triplicate.

### Screening for sesquiterpene lactones

To search sesquiterpenes lactones in the extracts, the Baljet reaction was used. A 1% picric acid (w/v) solution in ethanol and a 10% sodium hydroxide (w/v) aqueous solution were combined at a 1:1 ratio and added to 2–3 mg of sample. A positive reaction was indicated by an orange to red color change [15,16].

## Results and discussion

Plants used in the present study were selected according to their traditional usage for the treatment of tuberculosis or symptom-related diseases by the Sonoran ethnic groups. Table 1 shows the identification of the studied plants, by scientific and common name, traditional use, parts analyzed, and the ethnic group that uses them as medicinal remedy. The extraction method was chosen based on the consideration that methanolic extracts may contain a wide range of chemical compounds with biological activity such as terpenoids, phenols, flavonoids, saponines, steroids, and others [17-20]. In addition, some studies have reported that methanolic extracts are more active than aqueous extracts in their antibacterial activity [21].

Table 2 shows the MIC values of methanolic extracts of the plants studied. The methanolic extract from *Ambrosia confertiflora* showed a MIC value of 200 μg/mL against *M. tuberculosis* H37Rv. Methanolic extracts from *Ambrosia ambrosioides* and *Guaiacum coulteri* showed MICs of 790 and 1000 μg/mL, respectively. On the other hand, the extracts from *Acalypha californica*, *Schinus molle, Vallesia glabra, Baccharis glutinosa, Phoradendrom californicum* and *Acacia farnesiana* did not show inhibition even at the highest tested concentration. *Guaiacum coulteri*, traditionally used against tuberculosis by ethnics groups, resulted less effective *in vitro* than *A. confertiflora* (which is not traditionally indicated as anti-TB plant) in the anti-mycobacterial assay. However, as we use only flowers from *G. coulteri* for the anti-mycobacterial assay, it is important to evaluate other parts of this plant to assess this activity.

**Table 2:** ***In vitro *** **anti-mycobacterial activity of medicinal plant’s methanol extracts**

**Scientific name**	**Part of the plant used**	**MIC (μg/mL)**
*Ambrosia confertiflora* DC.	Aerial parts	200
*Ambrosia ambrosioides* (Cav.) W.W. Payne	Aerial parts	790
*Guaiacum coulteri* A. Gray	Flower	1000
*Acalypha californica* Benth.	Aerial parts	NI
*Schinus molle* L.	Leaf	NI
	Fruit	NI
*Vallesia glabra*	Aerial parts	NI
*Baccharis salicifolia* (Ruíz & Pavon) Pers.	Aerial parts	NI
*Phoradendrom californicum* Nutt.	Aerial parts	NI
*Acacia farnesiana* (L.) Wild	Leaf	NI
	Fruit	NI
Rifampicin		0.1171

MIC: minimal inhibitory concentration.

NI: no inhibition even at the highest concentration tested (1000 μg/mL).

*Ambrosia confertiflora* is used by Sonoran ethnic groups to treat diseases with symptoms closely related to tuberculosis, such as fever and lack of appetite (Table 1), and furthermore, the family to which it belongs (Asteraceae) contains species with a high concentration of sesquiterpene lactones (SQL) identified for their wide variety of biological activities, including their anti-mycobacterial effect [22,23]. Species of the genus *Ambrosia* have been previously reported for their anti-tuberculosis activity, associated to SQL [24]. It is likely that the anti-tuberculosis activity observed in *A. confertiflora* and *A. ambrosioides* is associated with the presence of such molecules.

*Acacia farnesiana*, *G. coulteri*, and *S. molle* have been referred by Sonoran traditional medicine as anti-tubercular agents, but only *G. coulteri* resulted active under the conditions tested. In this study we used leaves and fruits of *A. farnesiana* and *S. molle* to evaluate the anti-mycobaterial activity which could explain our results, especially in the case of *A. farnesiana* from which several products (gum, flower, seed, leaves, cortex, root) are used by ethnias as anti-tubercular medicine.

With the exception of *Schinus molle*, none of the plants studied had been evaluated before in Mexico for their anti-tuberculosis properties [25]. Molina-Salinas et al. [25] reported a MIC of 125 μg/mL for the hexane extract of *S. molle*; however, for the methanolic extract evaluated in the present study, no activity was detected even at the highest concentration tested (1000 μg/mL). The differences between the results obtained by Molina-Salinas et al. [25] and ours are attributed to the presence of mainly non-polar compounds in the hexane extract, in contrast with the methanolic extract where high polarity compounds are present.

Diverse methods and procedures to assess anti-mycobacterial agents are currently used, resulting in a diversity of cut-off values to define a MIC value as active: MIC ≤100 μg/mL, ≤125 μg/mL, ≤ 200 μg/mL [6,25-27]. For this reason, no international standard has yet been established to adequately define the level of anti-mycobacterial activity. For the present study we defined as active the extract with a MIC value of ≤ 200 μg/mL, considering the presence of the active component (s) at adequate concentration for their further isolation and purification, such was the case of *Ambrosia confertiflora* methanolic extract.

References to medicinal plants used by ethnic groups are mainly considered as a relevant guideline in the research of natural products; however, in many cases it is possible to validate the biological activity referred, but in others it is not possible to confirm scientifically their medicinal properties.

The difference in the anti-mycobacterial activity of *A. confertiflora*, *A. ambrosioides* and *G. coulteri* may be attributed to the difference in the relative concentration of the active compound in these plants. However, further research is required to demonstrate this statement.

Several reports correlate the high content of terpenoid compounds and different biological activities for the genus *Ambrosia*, such as anti-fungal, anti-trypanosomic, and anti-leishmania, as well as an anti-malaria effect [28-31].

From *A. confertiflora*, SQLs could be responsible for the anti-mycobacterial effect observed in the present study [32,33]. Cantrell et al. [23], in their review, reported plant-derived terpenoids and some synthetic analogs with variable anti-tubercular activity by the BACTEC 460 system. Particularly, over 50 sesquiterpene are reported [23] in that evaluation, where the most active compounds from *A. confertiflora* were santamarine and reynosin with a MIC value of 64 μg/mL. The MIC values obtained in our study are higher in comparison with those previously reported for reynosin and santamarine, since these are pure compounds, whereas ours were crude extracts.

Based on the latter and considering that the SQLs isolated by Yoshioka and collaborators are from a chloroform extract [32], we included less polar crude extracts such as chloroform, dichloromethane, and ethyl acetate from *A. confertiflora* and evaluated the effect on the growth of *M. tuberculosis* H37Rv. The results evidenced an increase in the activity of these extracts in comparison to the methanolic extract; but the main activity was obtained with chloroform with a MIC value of 90 μg/mL. On the other hand, the dichloromethane and ethyl acetate extracts showed MIC values of 120 and 160 μg/mL, respectively (Table 3). Highly polar molecules exhibit a reduced transport through the outer lipid layer of mycobacteria and, in consequence, lower anti-mycobacterial activity, whereas less polar molecules exhibit higher permeability [34,35]. These reports are in agreement with our results, where the extracts obtained with less polar solvents showed better activity than those exhibited by the methanolic extracts.

**Table 3:** ***In vitro *** **anti-mycobacterial activity results of organic extracts obtained from *Ambrosia confertiflora*, and sesquiterpene lactones status**

**Organic extracts**	**MIC (μg/mL)**	**Baljet reaction**
Chloroform	90	+
Dichloromethane	120	+
Ethyl acetate	160	+
Methanol	200	+
Rifampicin	0.1171	ND

MIC: minimal inhibitory concentration; ND: Not determined; +: positive.

Our findings reveal that the extraction method with intermediate-polarity solvents (e.g. dichloromethane) favors the possibility to obtain active compounds against *M. tuberculosis*, since it would be quite difficult to obtain them from aqueous extracts (high-polarity) as reported by Camacho-Corona and collaborators [27]; hence, the active compounds against this pathogen must be either less polar or even non-polar [25]. For this reason, the aqueous extraction method would not be the ideal method to obtain anti-mycobacterial compounds.

Based on studies reporting that both *A. confertiflora* and *A. ambrosioides* are rich in SQLs [22,23], we chose to perform a qualitative Baljet reaction to establish the possible presence of these molecules, resulting positive for all the different extracts (chloroform, dichloromethane, ethyl acetate and methanol) of *A. confertiflora* (Table 3) and the methanolic extract from *A. ambrosioides*. Although this reaction is also positive for cardiac glycosides and others containing α, β-unsaturated lactones, anti-mycobacterial activity has been attributed to this kind of molecules. Further studies are being performed on *A. confertiflora* to isolate the active compounds.

## Conclusions

*Ambrosia confertiflora* and *A. ambrosioides* were included in this study, despite not being used for the treatment of tuberculosis by local ethnic groups, and showed the best anti-mycobacterial *in vitro* activity.

The anti-mycobacterial activity of *Guaiacum coulteri* is consistent with the traditional use by Sonoran ethnic groups as anti-tuberculosis agent.

However, in the case of *Schinus molle* and *Acacia farnesiana* which are used against tuberculosis by these groups, no anti-mycobacterial activity was found, since only leaves and fruits were evaluated, but not gum, flower, seed, cortex or root as used by ethnias especially in the case of *A. farnesiana.*

For these reasons it is important to investigate a broader spectrum of medicinal plants in order to find compounds active against *Mycobacterium tuberculosis*.

## Competing interests

The authors declare that they have no competing interests.

## Author’s contributions

AGE, RERZ, EWCA, CAVC conceived the study, analyzed data, and drafted the manuscript. EWCA, MNN, ERB were involved in generation of organic extracts. EWCA carried out the biological assay. All authors have read and approved the final manuscript.

## Pre-publication history

The pre-publication history for this paper can be accessed here:

http://www.biomedcentral.com/1472-6882/13/329/prepub
